# 641. Limited Utility of Epstein-Barr Virus (EBV) Surveillance for Predicting Post-Transplant Lymphoproliferative Disorder in EBV Seropositive Adult Lung Transplant Recipients

**DOI:** 10.1093/ofid/ofad500.705

**Published:** 2023-11-27

**Authors:** Madeleine R Heldman, Yeh-Chung Chang, Jennifer Saullo, Arthur W Baker, Eileen K Maziarz, Patrick C Tam, Julia A Messina, John M Reynolds, Cameron R Wolfe, Barbara D Alexander

**Affiliations:** Duke University, Durham, North Carolina; Duke University, Durham, North Carolina; Duke University, Durham, North Carolina; Duke University School of Medicine, Durham, North Carolina; Duke University Medical Center, Durham, NC; Duke University School of Medicine, Durham, North Carolina; Duke University, Durham, North Carolina; Duke University School of Medicine, Durham, North Carolina; Duke University, Durham, North Carolina; Duke University School of Medicine, Durham, North Carolina

## Abstract

**Background:**

Post-transplant lymphoproliferative disorder (PTLD), which is often driven by Epstein-Barr virus (EBV), causes significant morbidity and mortality in lung transplant recipients (LTRs). EBV viremia surveillance with reduction of immunosuppression at certain viral load thresholds is a common strategy used to prevent PTLD or mitigate progression of PTLD in pre-clinical stages, but the utility of EBV surveillance in adult seropositive LTRs is poorly understood.

**Figure:**
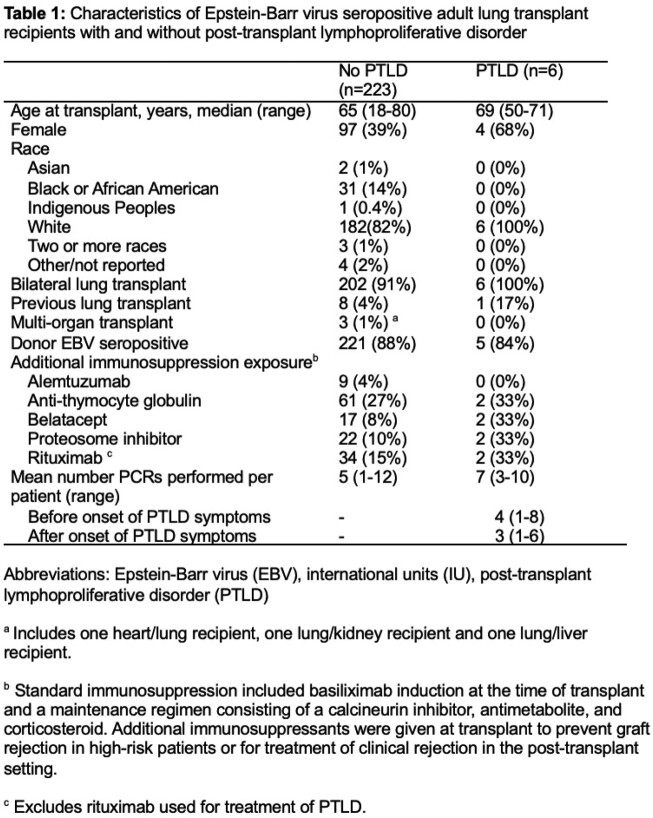

**Figure:**
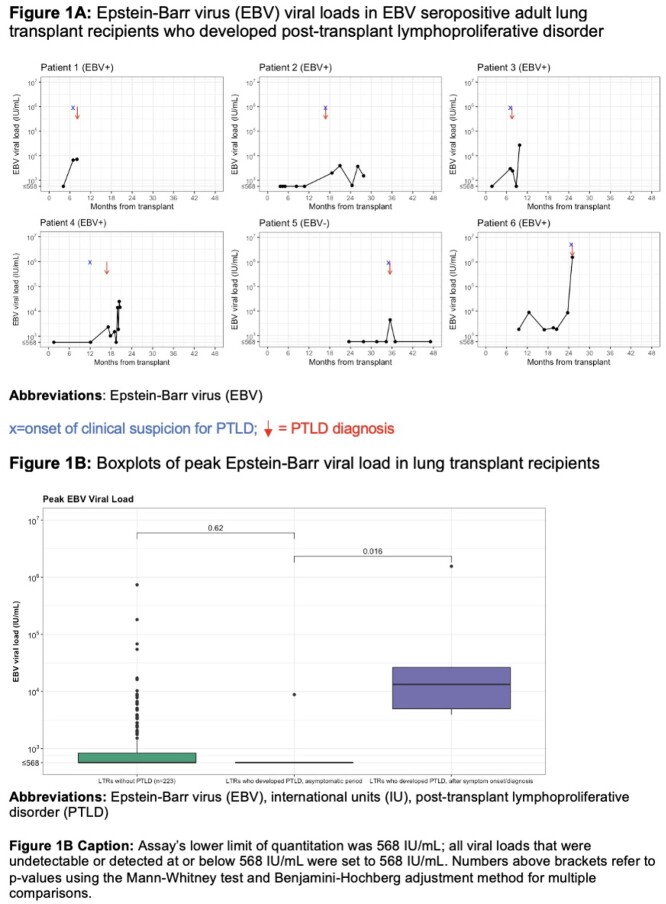

**Methods:**

In a retrospective cohort study of EBV seropositive adults who underwent lung transplant at a single academic center between 1/1/2019-12/31/2020, we compared peak whole blood EBV viral load (VL) among samples collected from 1) LTRs who developed PTLD, before PTLD symptom onset, 2) LTRs who developed PTLD, after PTLD symptom onset and 3) LTRs who did not develop PTLD. LTRs without any recorded EBV VLs were excluded. Patients were followed through 1/31/2023 (maximum 49 months post-transplant). Center protocol recommended quantitative EBV PCRs every 3 months between 3-18 months post-transplant, and as clinically indicated thereafter. Reduction of immunosuppression and evaluation for PTLD was at the discretion of clinicians.

**Results:**

Two-hundred twenty-nine LTRs with a total of 1095 EBV PCRs were included (40% female, median age 65 years at transplant, **Table 1**). Six (2.6%) LTRs developed biopsy-proven PTLD 8-35 months post-transplant (median 17 months); 5/6 PTLDs were EBV+. Clinical symptoms, rather than an asymptomatic high EBV VL, triggered evaluation for PTLD in all 6 cases. Only one of six LTRs with PTLD had an EBV VL above the assay’s lower limit of quantification before PTLD symptom onset (**Figure 1A**); among the other 5 LTRs, median time between most recent negative EBV PCR and PTLD symptom onset was 125 days (range 35-216). Peak EBV VL in LTRs with PTLD was significantly higher after PTLD symptom onset than in the asymptomatic period (p=0.016), but peak EBV VL was similar between asymptomatic LTRs who later developed PTLD and LTRs who did not develop PTLD (p=0.62, **Figure 1B**).

**Conclusion:**

In EBV seropositive adult LTRs , EBV surveillance rarely facilitated PTLD diagnosis and may not be an effective strategy for identifying asymptomatic individuals at high risk for future PTLD.

**Disclosures:**

**Arthur W. Baker, MD, MPH**, Insmed: Grant/Research Support|Medincell: Advisor/Consultant **Eileen K. Maziarz, MD**, Karius, Inc: Advisor/Consultant **Barbara D. Alexander, MD**, F2G Pharmaceuticals: Advisor/Consultant|HealthTrackRx: Advisor/Consultant|HealthTrackRx: Board Member|Leadiaint: Grant/Research Support|Merck: Advisor/Consultant|Scynexis: Grant/Research Support|Thermofisher: Advisor/Consultant

